# Pregnancy with a Severe Hemoglobinopathy: Unintended Consequences of Transfusions

**DOI:** 10.1155/2013/413502

**Published:** 2013-01-27

**Authors:** David Kim, Hector Mendez-Figueroa, Brenna L. Anderson

**Affiliations:** ^1^Department of Obstetrics and Gynecology, Women & Infants Hospital, The Warren Alpert Medical School of Brown University, Providence, RI 02905, USA; ^2^Maternal-Fetal Medicine Clinic, Department of Obstetrics and Gynecology, Women & Infants Hospital, The Warren Alpert Medical School of Brown University, 101 Dudley Street, 3rd Floor, Providence, RI 02905-2401, USA

## Abstract

We report a case of a pregnant woman with a complex hemoglobinopathy who developed a symptomatic anemia at 28 weeks of gestation and was treated with multiple transfusions of type-specific packed red blood cells. Shortly thereafter, she developed a fever and joint pains, along with laboratory values consistent with hemolysis. Timing suggested a delayed transfusion reaction. An extensive evaluation including red blood cell antigen identification and cross-reaction failed to reveal the cause for her hemolysis. Despite her critically low hemoglobin levels, her transfusions were withheld in an attempt to allow the patient to recover conservatively. With this strategy, her hemoglobin remained below her baseline, but her symptoms began to improve. Her laboratory values normalized, and hemolysis was no longer evident. Three weeks later, her hemoglobin levels returned back to her baseline without additional intervention. She went on to deliver a full-term male infant.

## 1. Introduction

Worldwide, approximately 270 million people are estimated to be either silent carriers or directly affected by a hemoglobin disorder. The incidence is on the rise, most notably in North America, reflecting a shift in the epidemiology [1]. Changes in population demographics due to increased ethnic integration have resulted in a higher number of infants born with a hemoglobinopathy, thus increasing the number of cases encountered in obstetrical practice [2]. This trend in shifting demographics has contributed to the increased complexity of these disorders, making them more difficult to manage. Whereas historical carriers would possess alterations on a single gene, patients with a variety of permutations are now becoming more frequent, resulting in a number of new phenotypes and challenging traditional guidelines [3, 4].

Treatment is especially challenging when patients become pregnant, as the physiologic demands of pregnancy place a larger burden on hemoglobin reserves. Pregnancy itself has also been identified as a known risk factor for developing hemolytic crises in patients with severe hemoglobinopathies [3, 5]. The American Society of Hematology currently recommends red blood cell transfusion to maintain patient's hemoglobin above 8.0-9.0 g/dL in patients with severe anemias [3]. For pregnant patients, many institutions employ more aggressive protocols aimed at maintaining hemoglobin levels above 10.0 g/dL and using transfusions as the first-line response to sustain adequate levels [4, 6, 7]. However, transfusions always carry risks, both immediate and delayed.

A hemolytic transfusion reaction is defined as the rapid destruction of red blood cells following a transfusion, and it occurs, in the United States, in 1 out of every 40,000 transfused units with incidence higher for those with repeat transfusions [6, 8]. We present a case of a severe hemoglobinopathy in pregnancy that was complicated by acute hemolysis and ultimately diagnosed as a delayed transfusion reaction. Conventional therapy produced worsening disease, and the patient was ultimately treated by withholding blood products. This case highlights the difficulty in treating pregnant patients with severe hemoglobinopathies, the need to consider other causes of anemia, and the need to contemplate different treatment options in such patients.

## 2. Case Report

A 22-year-old gravida 4 para 1 of Cambodian descent was followed at a high-risk clinic in this large, tertiary care, academic hospital secondary to a severe hemoglobinopathy. During childhood, she was diagnosed with a complex blood dyscrasia consisting of an HbE mutation on one beta chain and a double deletion on a single alpha gene in the CIS position together with an alpha Constant Spring mutation. Hemoglobin electrophoresis showed 78% hemoglobin-A, 3.5% hemoglobin-F, 14% hemoglobin-EA2, 2.7% hemoglobin BART, and 1.5% hemoglobin Constant Spring. Her baseline bilirubin ranged from 1.5 to 2.0 mg/dL with baseline hemoglobin of 7 g/dL and a reticulocyte count of 3.0 to 3.5%. She was known to have circulating anti-E antibodies at a stable 1 : 2 titer level. Though her disorder has been mostly stable requiring no regular interventions, she does have a history of multiple transfusions prompted by no identified cause at various points in her life without any reactions and none in the past several years prior to pregnancy. Her past pregnancy was complicated by intrauterine growth restriction and preterm birth at 35 weeks, and her hemoglobin levels remained at her baseline throughout gestation. She has history of asthma controlled with budesonide and was prescribed prenatal vitamins and folic acid as well as weekly injections of 17 alpha-hydroxyprogesterone caproate for prevention of recurrent preterm birth. Her surgical history included a cholecystectomy. She denied any tobacco, alcohol, or drug use and lived with the father of the baby. She had a family history of alpha thalassemia on both her maternal and paternal sides.

She presented for a routine visit at 26 weeks of gestation with complaints of cough and nasal congestion along with fatigue for the past week. Laboratory analysis revealed a hemoglobin level of 5.7 g/dL and a hematocrit of 26.1%. She was transfused with 2 units of type-specific cytomegalovirus (CMV) negative packed red blood cells (PRBCs) as an outpatient with no signs of an adverse reaction. Two weeks later, during her follow-up visit, she was again complaining of fatigue and palpitations. Her hemoglobin level at that point was 5.4 g/dL. She received two additional units of type-specific PRBC, although these units were inadvertently rapidly infused in less than 2 hours. A few hours later, she became more fatigued with associated body aches and pains. She developed a fever and reported dark urine along with complaints of nausea but no vomiting or changes in bowel movements. She denied any contractions, vaginal bleeding, loss of fluid, or changes in fetal movement. She was admitted with the presumed diagnosis of hemolysis secondary to a transfusion reaction. For the first three days of hospitalization, her hemoglobin continued to drop. Transfusion medicine was consulted, and 2 units of type-specific blood screened for multiple antibodies were transfused (Table 1). Her fevers mildly improved with acetaminophen but returned quickly with temperatures climbing to 103°F. Her joint pains continued along with dyspnea with a respiratory rate reaching 39/min. She developed mild tachycardia with her heart rate ranging between 100 and 120 beats per minute.

The patient was screened for various known immunogenic red blood cell antigens. CMV, *Babesia*, direct and indirect Coombs, antinuclear antibodies, and G6PD deficiency testing all returned negative. Testing performed at a reference laboratory revealed reactivity to Lea, S, K, and Fyb antigens; however, testing against record samples showed that the units she received were negative for the presence of these antigens. Although her values continued to worsen, a multidisciplinary discussion concluded that the patient was not to be transfused but rather managed expectantly. Her hemoglobin continued to fall and stabilized around 5.0 g/dL. Her bilirubin, notably her direct bilirubin, kept increasing, peaking on hospitalization day 5. Her jaundice was noticeably worse. However, after hospitalization day 6, her symptoms began to improve. Her fever subsided, and she remained afebrile throughout the rest of her hospital stay. Her joint pains disappeared, and her breathing returned to normal within 24 hours. Her fetus showed no signs of distress during the entire course of her hospital stay. 

On hospitalization day 8, she remained stable and asymptomatic. Her hemoglobin stabilized at around 5.0/dL, but her reticulocyte count improved demonstrating an adequate bone marrow response. In addition, her bilirubin and LDH levels fell, indicating resolving hemolysis. The clinical and laboratory improvement along with the patient's wishes led to the decision to discharge and continue with an outlined outpatient management scheme. Three weeks after discharge, her hemoglobin and bilirubin levels returned back to her original baseline. Her pregnancy remained uneventful, and she went on to spontaneously vaginally deliver a 2755 g male infant at 38 weeks and 0 day with APGAR scores 8 and 9 without any complications. Her hemoglobin level was 7.3/dL on labor admission and 6.1/dL at discharge. Both the newborn and her previous child have been referred to hemoglobinopathy screenings.

## 3. Discussion

The management of hemoglobinopathy in pregnancy is uniquely challenging because even if the maternal physiology has adapted to a lower hemoglobin baseline, the fetus may not tolerate it as well as the mother does. Severe anemia, defined as a maternal hemoglobin level lower than 6.0/dL, has been associated with placental insufficiency and abnormal fetal oxygenation, resulting in nonreassuring fetal heart rates, intrauterine growth restriction, fetal cerebral vasodilation, and fetal demise [9]. 

In addition, pregnancy increases the blood volume by 50%, placing further stress on already low levels, and the additional stress may be beyond what the adapted maternal physiology is able to handle, increasing maternal risk of neurologic damage, organ failure, and maternal mortality. The exact decision on when to transfuse varies based on the institution and practitioner. These concerns led to the initial transfusion recommendation in this patient who presented with hemoglobin levels around 5.0 g/dL in the late second trimester [9].

This report illustrates, though, that despite standard blood screening, there remains a risk with transfusions, even in a patient without previous reactions. When treating patients with severe anemia, the decision to transfuse should weigh the pros and cons of such intervention. Hypertransfusion increases the risk of iron overload, the development of alloantibodies, and the transmission of infections [6]. The risk of alloimmunization is particularly notable, as the rate has been estimated as high as 37% [6]. This risk is higher with donor-recipient ethnic disparity, another inevitable consequence of the changing demographics [2, 6].

This patient has a non-transfusion-dependent thalassemia, since she does not require regular transfusions for survival. Recent hematology research suggests that transfusing such patients can even be more harmful as their bodies become accustomed to functioning with extraordinarily low hemoglobin levels. However, this literature also acknowledges that pregnancy is a physiologic state that may necessitate a transfusion even in such patients [4]. The precipitating factor leading to hemolysis in our case was never identified, although it was possible that her viral illness could have led to her condition. 

Regardless of whether thalassemia is the predominant hemoglobinopathy, as in our case, or other severe blood dyscrasias are present, the decision to pursue transfusion should not be undertaken lightly. This decision should include both clinical and laboratory data [4, 7]. Standard cutoffs for transfusion are not sufficient, since they fail to consider different baseline hemoglobin levels at which patients are accustomed to as well as the changes resulting from physiological perturbations during pregnancy. 

It is important to note that although expectant management resulted in a favorable clinical outcome, a contingency plan had been outlined in the event that maternal or fetal deterioration developed which a conservative transfusion support strategy with a multidisciplinary input. Our overall reassurance was reinforced by the resolution of symptoms as well as the lack of fetal distress on testing. Although laboratory values failed to improve immediately, symptoms continued to resolve thus dictating our clinical conduct. Conservative management of pregnant patients remains an acceptable strategy, as long as the maternal and fetal health is constantly evaluated. A similar case to our patient had not previously been described in the available literature; however, the most similar—a number of case reports of delayed transfusion reactions in maternal sickle cell patients—suggests further caution in immediately pursuing transfusion support in these patients [10].

In conclusion, a careful examination of the clinical state of both the patient and fetus should be considered. All potential risks of a transfusion, especially repeat transfusions, need to be considered with the understanding that additional therapy may result in harm. A multidisciplinary team, with representation from medicine, hematology, and transfusion medicine if available, should be involved and actively participate in the patient care. If the decision is made to shift (or move) from active transfusion support to expectant management, additional units should still be typed, screened, and readily available in the event where the clinical situation rapidly deteriorates.

## Figures and Tables

**Table 1:** Important lab values from admission to hospital to 2 follow-up outpatient visits.

	Admission	Day 0	Day 1		Day 2		Day 3		Day 4	Day 5	Day 6	Day 7	Day 8	Outpatient-PD day 2	Outpatient-PD 3 weeks
Hb (g/dL)	8.3	6.0	7.7	7.0	5.8	8.2	6.5	6.4	5.7	5.3	5.4	5.2	5.1	5.4	6.8
Hematocrit (%)	26.1	19.0	23.6	21.2	18.2	24.8	19.9	19.4	17.4	16.0	16.9	16.7	16.6	18.0	23.3
WBC (K/*μ*L)	13.6	—	7.9	—	6.6	—	—	—	4.5	5.3	6.6	8.5	10.3	11.0	7.9
RBC (K/*μ*L)	3.85	—	3.29	—	3.38	—	—	—	2.53	2.39	2.39	2.61	2.59	2.78	3.56
Platelet (K/*μ*L)	222	—	159	—	154	—	—	—	168	171	157	236	198	250	249
MCV (fL)	68	—	71.6	—	73.4	—	—	—	68.9	66.9	66.1	64	64	64.6	65.5
RDW (%)	33.9	—	34.1	—	33.2	—	—	—	38.7	33.9	32.6	31.5	31.1	32.1	30.8
Retic count (%)	—	—	—	—	3.5	—	3.4	—	3.3	4.8	3.8	5.2	6.1	6.4	—
BUN (mg/dL)	16	—	14	—	11	—	—	—	—	—	—	—	—	—	—
Creatinine (mg/dL)	0.53	—	0.48	—	0.45	—	0.40	—	0.42	0.46	0.46	0.50	0.51	0.49	—
LDH (U/L)	635	—	624	—	803	—	763	—	809	904	865	838	829	470	—
Total bilirubin (mg/dL)	10.0	—	6.3	—	9.9	—	11.1	—	10.6	14.0	10.7	8.0	6.7	3.9	2.2
Direct bilirubin (mg/dL)	—	—	2.1	—	5.1	—	—	—	7.6	10.1	7.6	5.6	4.7	2.6	1.2
AST (U/L)	—	—	—	—	30	—	—	—	25	—	—	—	—	—	—
ALT (U/L)	—	—	—	—	9	—	—	—	9	—	—	—	—	—	—
Alk phos (U/L)	—	—	—	—	131	—	—	—	165	—	—	—	—	—	—
Temperature (°C)	38.2	36.1	38.1	36.8	36.8	39.3	37.2	37.0	37.0	37.1	36.8	36.7	36.6	37.1	—
Heart rate (bpm)	139	111	85	123	130	92	116	124	101	109	110	118	107	68	78
Respiratory rate (breaths/min)	16	23	20	30	39	9	16	20	18	20	18	16	20	15	—
Transfusions completed (packed RBCs)	—	2 units	—	—	2 units	—	—	—	—	—	—	—	—	—	—
